# Statin therapy is associated with improved survival in patients with ventricular tachyarrhythmias

**DOI:** 10.1186/s12944-019-1011-x

**Published:** 2019-05-24

**Authors:** Jonas Rusnak, Michael Behnes, Tobias Schupp, Siegfried Lang, Linda Reiser, Gabriel Taton, Armin Bollow, Thomas Reichelt, Dominik Ellguth, Niko Engelke, Uzair Ansari, Ibrahim El-Battrawy, Thomas Bertsch, Christoph A. Nienaber, Muharrem Akin, Kambis Mashayekhi, Christel Weiß, Martin Borggrefe, Ibrahim Akin

**Affiliations:** 1 First Department of Medicine, University Medical Centre Mannheim (UMM), Faculty of Medicine Mannheim, University of Heidelberg, European Center for AngioScience (ECAS), and DZHK (German Center for Cardiovascular Research) partner site Heidelberg/Mannheim, Mannheim, University of Heidelberg, Theodor-Kutzer-Ufer 1-3, 68167 Mannheim, Germany; 2 Institute of Clinical Chemistry, Laboratory Medicine and Transfusion Medicine, General Hospital Nuremberg, Paracelsus Medical University, Nuremberg, Germany; 3 Royal Brompton and Harefield Hospitals, NHS, London, UK; 4 Department of Cardiology and Angiology, Hannover Medical School, Hannover, Germany; 5 Clinic for Cardiology and Angiology II, Universitaetszentrum Freiburg Bad Krozingen, University of Freiburg, Bad Krozingen, Germany; 6 Institute of Biomathematics and Medical Statistics, University Medical Center Mannheim (UMM), Faculty of Medicine Mannheim, Heidelberg University, Mannheim, Germany

**Keywords:** Sudden cardiac death, Ventricular tachyarrhythmias, Ventricular fibrillation, Statin therapy

## Abstract

**Objectives:**

The study sought to assess the impact of statin therapy on survival in patients presenting with ventricular tachyarrhythmias.

**Background:**

Data regarding the outcome of patients with statin therapy presenting with ventricular tachyarrhythmias is limited.

**Methods:**

A large retrospective registry was used including all consecutive patients presenting with ventricular tachycardia (VT) or fibrillation (VF) from 2002 to 2016. Patients with statin were compared to patients without statin therapy (non-statin). The primary prognostic endpoint was long-term all-cause death at 3 years. Uni- and multivariable Cox regression analyses were applied in propensity-score matched cohorts.

**Results:**

A total of 424 matched patients was included. The rates of VT and VF were similar in both groups (VT: statin 71% vs. non-statin 68%; VF: statin 29% vs. 32%; *p* = 0.460). Statin therapy was associated with lower all-cause mortality at long-term follow-up (mortality rates 16% versus 33%; log rank, *p* = 0.001; HR = 0.438; 95% CI 0.290–0.663; *p* = 0.001), irrespective of the underlying type of ventricular tachyarrhythmia (VT/VF), left ventricular ejection fraction (LVEF) > 35%, presence of an activated implantable cardioverter defibrillator (ICD), cardiogenic shock or cardiopulmonary resuscitation (CPR).

**Conclusion:**

Statin therapy is independently associated with lower long-term mortality in patients presenting with ventricular tachyarrhythmias on admission.

**Trial registration:**

Clinicaltrials.gov, NCT02982473, 11/29/2016, Retrospectively registered.

## Condensed abstract

This study retrospectively examined the impact of a statin therapy on survival in 424 propensity-matched patients admitted with ventricular tachyarrhythmias. Presence of statin therapy was independently associated with lower long-term mortality (mortality rates 16% versus 32%; log rank *p* = 0.001; HR = 0.438; 95% CI 0.290–0.663; *p* = 0.001).

## Introduction

Epidemiological studies demonstrated a distinct association between cholesterol levels and all-cause as well as cardiovascular mortality [1–4]. Therefore, international guidelines recommend lipid-lowering therapy in patients at risk for and with already established cardiovascular diseases (CVD) as an effective treatment for secondary and primary prevention of atherosclerotic cardiovascular disease. Statins competitively inhibit HMG-CoA reductase activity and currently represent the first-line therapy in lowering lipid-levels [3, 5].

Large, randomized controlled trials (RCT) in patients with acute myocardial infarction (AMI) or coronary artery disease (CAD) demonstrated that lipid lowering therapy by statins (i.e. simvastatin or pravastatin), was associated with a reduced all-cause and cardiovascular mortality at five to six years [6, 7]. Furthermore, statin therapy was also investigated as an adjunct therapy in patients with chronic heart failure due to any cause. However, within the RCTs, namely GISSI-HF and CORONA, the use of rosuvastatin was not associated with a reduction of mortality or sudden cardiac death (SCD) at three to four years [8, 9]. The authors based their study concept on the multiple mechanisms of statins beyond prevention in artheroslerosis and therefore included any kind of heart failure patients without any strict inclusion criteria independently of cholesterol cutoffs for the use of rosuvastatin [10].

The multiple mechanisms of statins beyond lipid-lowering have been described recently and may include additional anti-arrhythmic effects. Statins stabilize the atherosclerotic and ischemic burden, which might indirectly translate into anti-arrhythmic effects inhibiting the onset of ventricular tachyarrhythmias and SCD. A direct antiarrhythmic effect apart from affecting arterosclerosis has also been suggested [11, 12]. Several observational studies investigating mostly patients with heart failure showed a reduction of rates of ventricular tachycardia (VT), ventricular fibrillation (VF) and SCD, as well as reduced rates of appropriate ICD-therapy, which might in turn impact secondary reduction of mortality due to ventricular tachyarrhythmias [10, 13–20]. However, no data is available at present, whether statins may impact secondary long-term survival in patients after presenting with ventricular tachyarrhythmias on admission.

Therefore, the present study evaluates the prognostic impact of statin therapy on long-term survival in patients presenting with ventricular tachyarrhythmias on admission.

## Methods

### Study patients, design and data collection

The present study retrospectively included all consecutive patients presenting with ventricular tachyarrhythmias on hospital admission from 2002 until 2016 at the First Department of Medicine, University Medical Centre Mannheim, Germany. Using the hospital information system, all relevant clinical data related to the index event were documented. The data, analytic methods, and study materials will be made available to other researchers for purposes of reproducing the results or replicating the procedure onreasonable personal request to the corresponding author.

Ventricular tachyarrhythmias comprised VT and VF, as defined by current international guidelines [21]. Sustained VT was defined by duration of more than 30 s or causing hemodynamic collapse within 30 s. Non-sustained VT was definded by duration of less than 30 s both with wide QRS complex (≥120 milliseconds) at a rate greater than 100 beats per minute [21]. Ventricular tachyarrhythmias were documented by 12-lead ECG (electrocardiography), ECG tele- monitoring, ICD or in case of unstable course or during resuscitation pulmonary resuscitation (CPR) by external defibrillator monitoring. Documented VF was treated by external defibrillation and in case of prolonged instability with additional intravenous anti-arrhythmic drugs during CPR.

Further data being documented contained baseline characteristics, prior medical history, prior medical treatment, length of index stay, detailed findings of laboratory values at baseline, data derived from all non-invasive or invasive cardiac diagnostics and device therapies, such as coronary angiography, electrophysiological examination, data being derived from prior or newly implanted cardiac devices, including those ICD already implated at index and at follow-up, pacemakers or cardiac contractility modulation (CCM), as well as imaging modalities, such as echocardiography or cardiac magnetic resonance imaging (cMRI).

Every revisit at the outpatient clinic or re-hospitalization was documented when related to recurrent ventriculartachyarrhythmias and adverse cardiac events. Adverse cardiac events comprised acute heart failure, CPR, cardiac surgery, recurrent percutaneous coronary intervention, new implants or upgrades of cardiac devices, and worsening or improvement of left ventricular function.

Documentation period lasted from index event until 2016. Documentation of all medical data was performed by independent cardiologists at the time of the patients’ individual period of clinical presentation, being blinded to final data analyses.

The present study is derived from an analysis of the “Registry of Malignant Arrhythmias and Sudden Cardiac Death - Influence of Diagnostics and Interventions (RACE-IT)” and represents a single-center registry including consecutive patients presenting with ventricular tachyarrhythmias, being acutely admitted to the University Medical Center Mannheim (UMM), Germany (clinicaltrials.gov identifier: NCT02982473) from 2002 until 2016. The registry was carried out according to the principles of the declaration of Helsinki and was approved by the medical ethics commission II of the Faculty of Medicine Mannheim, University of Heidelberg, Germany.

The medical center covers a general emergency department for emergency admission of traumatic, surgical, neurological and cardiovascular conditions. Interdisciplinary consultation is an inbuilt feature of this 24/7 service, and connects to a stroke unit, four intensive care units (ICU) with extracorporeal life support and a chest pain unit (CPU) to alleviate rapid triage of patients. The cardiologic department itself includes a 24 h catheterization laboratory, an electrophysiologic laboratory, a hybrid operating room and telemetry units.

### Index events, risk stratification measures and prognostic outcome

For the present study, all patients surviving index hospitalization after presenting with ventricular tachyarrhythmias between 2002 and 2016 were included. Each patient was counted only once for inclusion when presenting with the first episode of ventricular tachyarrhythmias. Indication to treat patients with statins was based on European guidelines of statin therapy [3]. Overall exclusion criteria comprised patients without complete follow-up data regarding mortality.

The primary prognostic endpoint was all-cause mortality during the follow-up period until 2016. All-cause mortality was documented using our electronic hospital information system and by directly contacting state resident registration offices (“bureau of mortality statistics”) across Germany. Identification of patients was verified by name, surname, day of birth and registered living address. In 48 patients, no data on patients’ survival could have been retrieved, as those patients were not even reachable by telephone, and were therefore excluded from final analyses (corresponding lost to follow-up rate of 1.7%).

### Statistical methods

Quantitative data are presented as mean ± standard error of mean (SEM), median and interquartile range (IQR), and ranges depending on the distribution of the data and were compared using the Student’s *t* test for normally distributed data or the Mann-Whitney *U* test for nonparametric data. Deviations from a Gaussian distribution were tested by the Kolmogorov-Smirnov test. Spearman’s rank correlation for nonparametric data was used to test univariate correlations. Qualitative data are presented as absolute and relative frequencies and compared using the Chi^2^ test or the Fisher’s exact test, as appropriate.

The following analyses were applied stepwise to evaluate the prognostic impact of statin therapy on all-cause mortality:

Propensity score analyses were performed, since this study includes consecutively all patients with ventricular tachyarrhythmias without randomization [22, 23]. Accordingly, propensity scores (probability for belonging to statin = yes) were calculated for each individual based predefined variables (see below). Afterwards, matched pairs were created using the method of nearest neighbor matching with a caliper distance of 5%. This means: each pair consisted of one individual with statin = yes and statin = no, whose propensity scores differed by less than 5%. We found 212 pairs with mean propensity score 0.5931 +/− 0.3113 (statin therapy = 0) and 0.6065 +/− 0.3121 (statin therapy = 1).

Uni-variable stratification was performed using the Kaplan-Meier method with comparisons between groups using uni-variable hazard ratios (HR) given together with 95% confidence intervals, according to the presence of a statin therapy within the propensity-matched cohorts.

Multivariable Cox regression models were developed using the “forward selection” option, where only statistically significant variables (*p* < 0.05) were included and analyzed simultaneously (see below). Multivariable Cox regressions were applied in the propensity-matched cohorts.

Predefined variables being used for propensity score matching (step A) and multivariable Cox-regressions (step C) included: baseline parameters (age, gender), chronic diseases (diabetes, chronic kidney disease (glomerular filtration rate < 90 mL/min/1. 73m^2^), left ventricular dysfunction), acute comorbidities (cardiogenic shock, cardiopulmonary resuscitation (CPR), acute myocardial infarction), presence of an implanted cardiac defibrillator (ICD), and underlying ventricular tachyarrhythmia (i.e. VT/VF) on admission.

Follow-up periods for evaluation of all-cause mortality were set at 3 years (=long-term), according to the median survival of statin patients to guarantee complete survival of at least 50% of patients. Patients not meeting long-term follow-up were censored.

The result of a statistical test was considered significant for *p* < 0.05, and a statistical trend for *p* < 0.10. SAS, release 9.4 (SAS Institute Inc., Cary, NC, USA) and SPSS (Version 25, IBM, Armonk, New York) were used for statistics.

## Results

### Study population

The propensity-matched cohort of consecutive patients surviving ventricular tachyarrhythmias on admission at our institution consisted of a total of 424 patients, of which each half was treated either with or without statins. The cohort was well matched for age, gender, arterial hypertension and diabetes. Statin patients revealed a higher rate of hyperlipidaemia, smoking and prior history of myocardial infarction. Overall, most patients presented with VT compared to VF with equally distributed rates in each group (VT: 71% versus 68%; VF: 29% versus 32%) (Table 1).

**Table 1:** Baseline characteristics and comorbidities after propensity score matching

Characteristic	Non statin (*n* = 212; 50%)		statin (*n* = 212; 50%)		*p* value
Age, median (range)	69 (16–92)		68 (25–86)		0.669
Gender, n (%)					
Male	159	(75)	164	(77)	0.569
Ventricular tachyarrhythmias, n (%)					
Ventricular tachycardia	144	(68)	151	(71)	0.460
Ventricular fibrillation	68	(32)	61	(29)	
Prior medical history, n (%)					
Chronic heart failure	71	(34)	72	(34)	0.918
Coronary artery disease	102	(48)	102	(48)	1.000
Myocardial infarction	49	(23)	67	(32)	**0.050**
Prior PCI	41	(19)	58	(27)	0.051
Prior CABG	25	(12)	43	(20)	**0.017**
Inflammatory heart disease	2	(0.9)	3	(1)	1.000
Valvular heart disease	30	(14)	21	(10)	0.179
Implanted cardiac devices					
ICD	40	(19)	38	(18)	0.802
Pacemaker	10	(5)	9	(4)	0.814
Ablation therapy	10	(5)	4	(2)	0.103
Stroke	23	(11)	25	(12)	0.759
Chronic kidney disease	106	(50)	103	(49)	0.771
Liver cirrhosis	3	(1)	1	(0.5)	0.623
COPD/asthma	29	(14)	21	(10)	0.228
Cardiovascular risk factors, n (%)					
Arterial hypertension	124	(59)	141	(67)	0.088
Diabetes mellitus	56	(26)	55	(26)	0.912
Hyperlipidemia	41	(19)	101	(48)	**0.001**
Smoking	51	(24)	72	(34)	**0.025**
Cardiac family history	25	(12)	22	(10)	0.643
Comorbidities at index stay, n (%)					
Acute myocardial infarction	25	(12)	35	(17)	0.164
STEMI	3	(1)	9	(4)	0.079
NSTEMI	22	(10)	26	(12)	0.540
Cardiogenic shock	21	(10)	18	(9)	0.614
Atrioventricular block	8	(4)	5	(2)	0.398
Cardiomyopathy	36	(17)	36	(17)	1.000
Hyperkalemia	1	(0.5)	2	(0.9)	1.000
Hypokalemia	18	(9)	16	(8)	0.721
Stroke	9	(4)	8	(4)	0.804
Intracranial hemorrhage	0	(0)	1	(0.5)	1.000
Clinically significant bleeding	11	(5)	1	(0.5)	**0.003**
Anemia	20	(9)	6	(3)	**0.005**
Septic shock	5	(2)	0	(0)	0.061
Cardiac surgery	4	(2)	7	(3)	0.359
Inadequate ICD shock	2	(0.9)	2	(0.9)	1.000
Atrial fibrillation	84	(40)	70	(33)	0.157
Paroxysmal	55	(26)	43	(20)	0.135
Persistent	9	(4)	3	(1)	
Permanent	20	(9)	24	(11)	
Asystole	5	(2)	9	(4)	0.277
PEA	2	(0.9)	0	(0)	0.499

CABG, coronary artery bypass grafting; COPD, chronic obstructive pulmonary disease; ICD, implantable cardioverter- defibrillator; NSTEMI, non-ST segment elevation myocardial infarction; PCI, percutaneous coronary intervention; PEA, pulseless electrical activity; STEMI, ST segment myocardial infarction. Bold type indicates *p* < 0.05

Target dosages were reached already at discharge, including simvastatin as the most frequently administered statin (*n* = 105; 50%; mean dosage 29 mg per day) followed by atorvastatin (*n* = 86; 41%; mean dosage 40 mg per day), fluvastatin (n = 10; 5% mean dosage 56 mg per day) and pravastatin (*n* = 8; 4%; mean dosage 21 mg per day) (data not shown).

Notably, no differences were found in both groups regarding prognosis-relevant comorbidities, including AMI, cardiogenic shock, cardiomyopathy, PEA (pulseless electrical activity) or asystole. Statin patients suffered less often from clinical significant bleeding and anaemia (Table 1).

### Cardiac diagnostics and therapies

As shown in Table 2, notably no differences were found in both groups regarding LVEF, rates of CPR, external defibrillation, fibrinolysis and TTM (targeted temperature management). Additionally, no differences were observed regarding extend of CAD and PCI rates, rates of electrophysiological testing and device therapy. CABG (coronary artery bypass graft) was more common in statin patients, alongside with higher rates of treatment with beta-blocker, ACEi (angiotensin-converting-enzyme inhibitor), ASA (acetylsalicylic acid), and dual antiplatelet therapy (Table 2).

**Table 2:** Cardiac diagnostics, therapies and survival data after propensity score matching

Characteristic	No statin (*n* = 212; 50%)		statin (*n* = 212; 50%)		*p* value
Left ventricular ejection function, n (%)					
LVEF ≥55%	63	(30)	60	(28)	0.352
LVEF 54–35%	63	(30)	62	(29)	
LVEF < 35%	86	(41)	90	(43)	
Cardiac therapies at index, n (%)					
Cardiopulmonary resuscitation	61	(29)	51	(24)	0.271
In hospital	26	(12)	21	(10)	0.439
Out of hospital	35	(17)	30	(14)	0.500
External defibrillation	59	(28)	51	(24)	0.375
External cardioversion	13	(6)	9	(4)	0.381
Systemic thrombolysis	7	(3)	5	(2)	0.558
Targeted temperature management (TTM)	5	(2)	8	(4)	0.398
Coronary artery disease, n (%)					
Coronary angiography, overall	131	(62)	140	(66)	0.363
Coronary artery disease, n (%)					
No evidence of CAD	47	(36)	41	(29)	0.425
1-vessel	24	(18)	33	(24)	
2-vessel	27	(21)	24	(17)	
3-vessel	33	(25)	42	(30)	
CTO	32	(24)	27	(19)	0.305
Presence of CABG	15	(12)	30	(21)	**0.027**
Intracoronary thrombus	2	(2)	6	(4)	0.180
CPR during coronary angiography	4	(3)	3	(2)	0.715
PCI, n (%)	28	(21)	43	(31)	0.081
Target lesions					
RCA	11	(5)	16	(8)	0.320
LMT	1	(0.5)	0	(0)	1.000
LAD	15	(7)	18	(9)	0.587
RIM	0	(0)	0	(0)	–
LCX	6	(3)	12	(6)	0.148
Bypass graft	0	(0)	3	(1)	0.248
Electrical therapies at index, n (%)					
Electrophysiological examination	72	(34)	88	(42)	0.109
Catheter ablation	18	(9)	19	(9)	0.863
Newly implanted devices at index, n (%)					
ICD	54	(26)	54	(26)	1.000
CRT-D	3	(1)	8	(4)	0.127
CRT-P	1	(0.5)	0	(0)	1.000
Pacemaker	4	(2)	2	(0.9)	0.685
Cardiac contractility modulation (CCM)	0	(0)	0	(0)	–
Subcutaneous ICD	0	(0)	1	(0.9)	1.000
Overall implanted devices	124	(59)	121	(57)	0.768
Medication at discharge, n (%)					
Beta-blocker	162	(76)	187	(88)	**0.001**
ACE-inhibitor	115	(54)	153	(72)	**0.001**
AT1-Antagonist	21	(10)	29	(14)	0.249
Aldosteron-antagonist	22	(10)	33	(16)	0.112
Aspirin only	58	(27)	78	(37)	**0.037**
Thienopyridine only	4	(2)	10	(5)	0.103
Dual antiplatelet therapy	25	(12)	52	(25)	**0.001**
Vitamin k antagonist	54	(26)	48	(23)	0.495
NOAC	7	(3)	2	(0.9)	0.175
Amiodarone	54	(26)	38	(18)	0.059
Digitalis	33	(16)	38	(18)	0.515
Hospitalization time, days, (median (IQR))					
Total hospitalization time	16 (8–27)		14 (8–26)		**0.009**
ICU time	3 (0–11)		2 (0–7)		**0.004**
Follow-up time, days, (mean; median (range))	1849; 1806 (25–5095)		1853; 1675 (18–5091)		**0.013**
All cause-mortality at 3 years, n (%)	70	(33)	33	(16)	**0.001**

CABG; coronary artery bypass grafting; CAD, coronary artery disease; CRT-D, cardiac resynchronization therapy plus defibrillator; CRT-P, cardiac resynchronization therapy plus pacemaker; CTO, chronic total occlusion; LAD, left anterior descending; LCX, left circumflex; LVEF, left ventricular ejection function; ICD, implantable cardioverter-defibrillator; ICU, intensive care unit; IQR, interquartile range; NOAC, new oral anticoagulants; PCI, percutaneous coronary intervention; RCA, right coronary artery; RIM, Ramus intermedius. Bold type indicates *p* < 0.05

### All-cause mortality and survival data

**Fig. 1:**
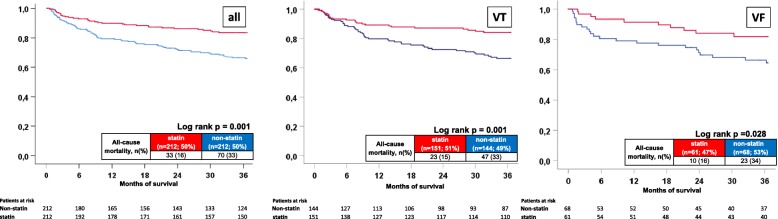
Overall all-cause mortality comparing statin with non statin patients (first panel), according to the underlying ventricular tachyarrhythmias, VT (second panel) and VF (third panel)

At long-term follow up (median 3.0 years (IQR 638 days – 2869 days), statin patients had significantly better survival compared to non-statin patients (long-term mortality rates 16% versus 33%; log rank *p* = 0.001; HR = 0.438; 95% CI 0.290–0.663; *p* = 0.001); (Fig. 1, left panel). Focusing on the presence of ventricular tachyarrhythmias, the prognostic benefit of statin patients was irrespective of the presence of VT (mortality rates 15% versus 33%; log rank *p* = 0.001; HR = 0.439; 95% CI 0.267–0.723; *p* = 0.001) (Fig. 1, middle panel) or VF (mortality rates 16% versus 34%; log rank *p* = 0.028; HR = 0.445; 95% CI 0.212–0.935; *p* = 0.032) (Fig. 1, right panel). Accordingly, long-term survival was not statistically different in statin patients presenting with VF compared to VT (mortality rates 16% versus 15%; log rank *p* = 0.796) (data not shown).

**Fig. 2:**
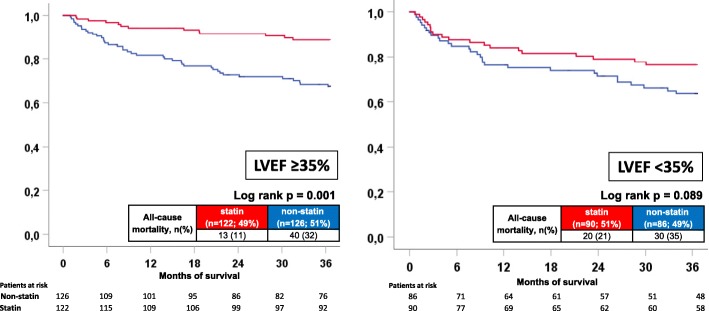
All-cause mortality comparing statin with non-statin patients according to LVEF ≥35% (left) and LVEF < 35% (right)

The prognostic benefit of statin patients was still evident when stratifying according to left ventricular ejection fraction (LVEF) above or below 35% (mortality rates: LVEF ≥35, 11% vs 32%, log-rank *p* = 0.001, HR = 0.302, 95% CI = 0.162–0.565, *p* = 0.001; LVEF < 35, 21% vs 35%; log rank *p* = 0.089) (Fig. 2, left and right panel).

**Fig. 3:**
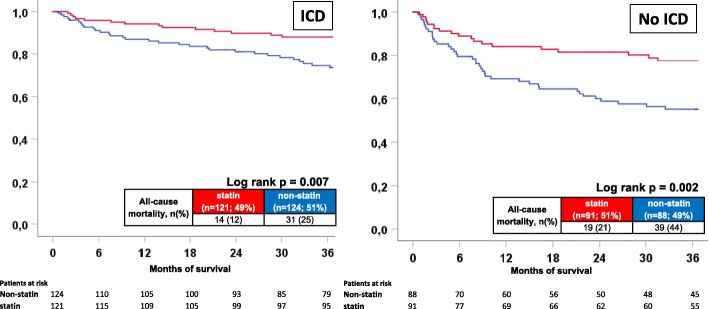
All-cause mortality comparing statin with non-statin patients according to the presence (left) or absence of activated ICD (right)

The presence of an activated ICD was associated with a comparable lower subsequent mortality both in statin (mortality rates: statin patients, 12% vs 21%, log rank *p* = 0.040, HR = 0.493, 95% CI =0.247–0.2983, *p* = 0.045) and non-statin patients (mortality rates: non-statin patients, 25% vs 44%, log rank *p* = 0.002, HR = 0.490, 95% CI = 0.305–0.785, *p* = 0.003) (data not shown). Furthermore, irrespective of the presence or absence of activated ICD mortality was still lower in statin compared to non-statin patients (mortality rates: ICD carriers, 12% vs 25%, log-rank *p* = 0.007, HR = 0.429, 95% CI = 0.227–0.804, *p* = 0.008; no ICD carrier, 21% vs 44%, log-rank p = 0.002, HR = 0.439, 95% CI = 0.252–0.754, p = 0.003) (Fig. 3, left & right panel).

### Multivariable cox models

**Fig. 4:**
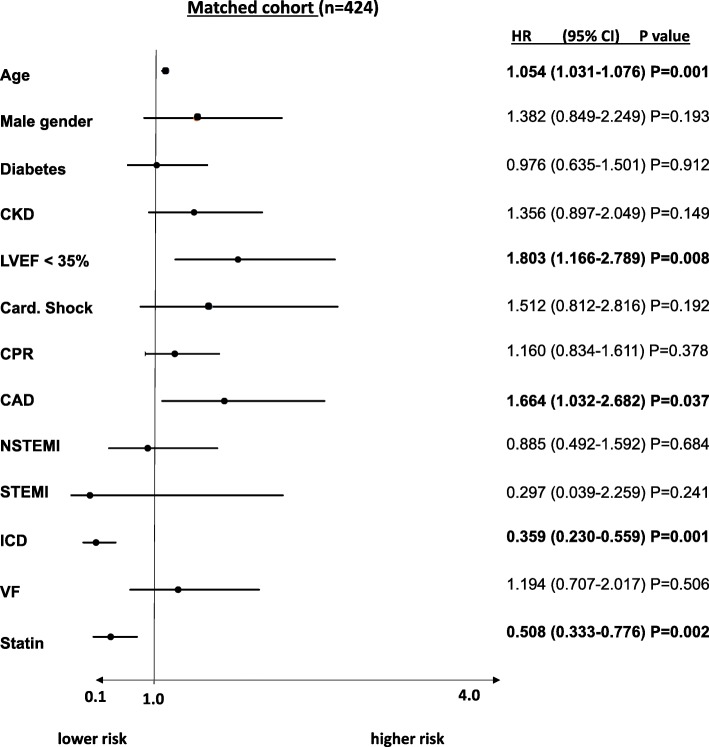
Statin therapy was still associated with beneficial survival even after adjusting for several prognosis-relevant factors

In multivariable Cox regression analysis, statin therapy was still associated with a lower risk of all-cause mortality in patients presenting with ventricular tachyarrhythmias (HR 0.508; CI 0.333–0.776, *p* = 0.002), comparable to the presence of an activated ICD (HR 0.359, 95% CI 0.359; 95% CI 0.230–0.559; *p* = 0.002). Furthermore, patients’ age (HR 1.054), male gender (HR 1.382), CKD (HR 1.356), LVEF < 35% (HR 1.803) and cardiogenic shock (HR 1.512) were significantly associated with an increased risk of long-term all-cause death. (Fig. 4).

## Discussion

The present study evaluates the prognostic impact of statin therapy on long-term survival in high-risk patients surviving ventricular tachyarrhythmias on admission.

This real-world data suggests that the presence of statin therapy is associated with a reduced risk of long-term all-cause mortality. The prognostic benefit of a statin therapy was evident irrespective of the underlying type of ventricular tachyarrhythmias (VT or VF), LV dysfunction or presence of an activated ICD. Additionally, the prognostic benefit associated with statin therapy was still evident even after controlling for patients’ age, gender, diabetes, CKD, AMI, CAD, cardiogenic shock, CPR, LVEF < 35% and presence of an activated ICD.

This study consistently identifies the presence of a statin therapy as a robust predictor of improved survival in patients surviving malignant arrhythmia on hospital admission. The major strength of the present study consists in the consecutive recruitment of patients with ventricular tachyarrhythmias and documented statin therapy straight from the admission scenario.

Several RCT and observational studies were able to demonstrate that lipid lowering therapy, especially by the use of statins, might lower all-cause mortality and cardiovascular death in patients with ischemic cardiomyopathy or CAD [6, 7, 14, 15, 18]. Statins may also prevent future SCD, reduce recurrences of ventricular tachyarrhythmias and improve overall survival in patients suffering from heart failure patients with LVEF < 30% [10, 15]. Notably, there is only one RCT published by Vrtovec et al. demonstrating that treatment with atorvastatin was able to significantly reduce the incidence of future SCD in patients with LVEF < 30% and cholesterol levels > 150 mg/dl at one year of follow-up [24]. Furthermore, several observational sub-studies of randomized ICD/CRT trials (MADIT II, MADIT-CRT, DEFINITE) demonstrated improved survival and reduction of arrhythmic deaths in patients treated with statins [10, 18, 19]. For instance, a sub-study of the MADIT-CRT trial demonstrated a significant reduction of appropriate ICD-therapies at 4 years in patients suffering from both ischemic and non-ischemic cardiomyopathy in the presence of statin therapy. Unfortunately, no information was given on the types and dosages of the administered statins [10]. Another sub-study of the DEFINITE trial found a reduced rate of premature beats, non-sustained VT and arrhythmic deaths in ICD carriers with non-ischemic cardiomyopathy with statin therapy [19]. However, the major limitation of these sub-studies is related to their preselected study cohorts and their unmatched character regarding the use of statins, which finally lead to further conflicting results in the according meta-analyses [12, 13, 17].

The following pathophysiological concepts may explain the beneficial effects of statin therapy in ventricular tachyarrhythmias and SCD, which may lay beyond the sole reduction of the atherosclerotic or ischemic burden. Statins are inhibitors of the HMG-CoA reductase and do reveal further pleiotropic pharmacological effects. These comprise prevention of ischemia, primary antiarrhythmic and anti-inflammatory effects [25]. The prevention of ischemia by statins was recently attributed to anti-coagulant and anti-thrombogenic effects, which in turn may reduce coronary micro-embolisms potentially causing myocardial ischemia and alleviating the development of ventricular tachyarrhythmias and SCD [25–28]. The anti-inflammatory effects of statins are represented mainly by their direct influence on atherosclerosis development. Inflammation itself represents a relevant causal factor for arrhythmogenesis [25, 29]. Within a community-based RCT including 1702 patients without evidence of CVD, statins were shown to reduce elevated CRP levels (C-reactive protein), which may indirectly lead to reduced risk of SCD [30, 31]. Furthermore, statins reveal direct anti-arrhythmic effects [32]. The antiarrhythmic properties were observed in experimental mouse models, where statins attenuated T-wave and Ca2+ alternans in isolated ventricular cardiomyocytes of mice treated with statin [33]. Both electrical disorders are precursors for an unstable electrical milieu alleviating the onset of malignant ventricular arrhythmia [23–25].

The use of statins is usually investigated in clinical studies investigating preselected patient populations, such as CAD, AMI, chronic heart failure or hyperlipidaemia, which sets the present study in clear contrast to the currently available evidence. The present study is based on an unselected all-comers design, thereby including “real-world” patients consecutively presenting on admission with life-threatening ventricular tachyarrhythmias, and still demonstrated beneficial effects for prognosis associated with statin therapy in this “high-risk” cohort. The findings were consistently and robustly demonstrated by applying stepwise stratification within a propensity score matched analysis. Underlying pathologies were strictly matched for included AMI (14%), CAD (68%), cardiogenic shock (10%), cardiomyopathy (17%) and atrial fibrillation (36%).

There is only one study by De Sutter et al., which is widely comparable to the data of the present study. Here, CAD patients presenting at index with life-threatening ventricular tachyarrhythmias were included following secondary implantation of an ICD with a mean follow-up of 490 days [34]. Only those patients with lipid lowering therapy (defined as treatment with both statins and fibrates) revealed less recurrences of ventricular tachyarrhythmias as documented by application of anti-tachycardia pacing (ATP) or shocks. The study did not exclude patients with normal or mildly reduced LVEF. Accordingly, the present study delivers evidence that statins may reveal beneficial effects also in high-risk patients with ventricular tachyarrhythmias with a still preserved left ventricular ejection fraction (LVEF > 55, 30% in each matched group).

Therefore, the present results support the hypothesis that statins may reveal several pleiotropic effects including potential anti-arrhythmic properties, since a significant reduction of secondary all-cause mortality was demonstrated in unselected but well-matched all-comer patients presenting with ventricular tachyarrhythmias on hospital admission when treated with statins.

### Study limitations

This observational and retrospective registry-based analysis reflects a realistic picture of consecutive health-care supply of high-risk patients presenting with ventricular tachyarrhythmias. Lost to follow-up rate regarding the evaluated endpoint of all-cause mortality was minimal. Additionally, heterogeneity within the study population was controlled by a stepwise statistical approach including multivariable adjustment for several important comorbidities and risk factors within a propensity matched cohort. Patients not surviving out of hospital CPR and not being transferred to the heart centre were not included in this study. All clinical data was documented reliably by individual cardiologists and specialists in internal medicine and cardiology during routine clinical care being blinded to final data analyses, alleviating the use of an independent clinical event committee.

## Conclusions

Statin therapy is associated with decreased long-term all-cause mortality in patients presenting with ventricular tachyarrhythmias.
